# Determination of 40 Elements in Powdered Infant Formulas and Related Risk Assessment

**DOI:** 10.3390/ijerph18105073

**Published:** 2021-05-11

**Authors:** Maria Luisa Astolfi, Daniela Marotta, Vittoria Cammalleri, Elisabetta Marconi, Arianna Antonucci, Pasquale Avino, Silvia Canepari, Matteo Vitali, Carmela Protano

**Affiliations:** 1 Department of Chemistry, “Sapienza” University of Rome, 00185 Rome, Italy; marialuisa.astolfi@uniroma1.it; 2 Department of Public Health and Infectious Diseases, “Sapienza” University of Rome, 00185 Rome, Italy; daniela.marotta@uniroma1.it (D.M.); vittoria.cammalleri@uniroma1.it (V.C.); arianna.antonucci@uniroma1.it (A.A.); matteo.vitali@uniroma1.it (M.V.); 3 National Research Council (CNR), Institute of Atmospheric Pollution Research, Via Salaria Km. 29.300, Monterotondo St., 00015 Rome, Italy; elisabetta.marconi@iia.cnr.it; 4 Department of Agricultural, Environmental and Food Sciences (DiAAA), University of Molise, via F. De Sanctis, 86100 Campobasso, Italy; avino@unimol.it; 5 Department of Environmental Biology, “Sapienza” University of Rome, 00185 Rome, Italy; silvia.canepari@uniroma1.it

**Keywords:** powdered infant formula, inorganic elements, inductively coupled plasma mass spectrometry, daily intake, health risk assessment

## Abstract

The aim of the study was to analyze all powdered infant formulas authorized and commercialized in Italy at the time of the study to measure the concentrations of 40 elements, and to estimate the infants’ intake of some toxic heavy metals for assessing possible related health risks. For this purpose, an optimized multi-element method was used through inductively coupled plasma mass spectrometry. Be, B, Al, Zr, Nb, Sb, Te, W, V, Cr and As concentrations were <LOD in more than 30% of samples. The levels of the other elements resulted to be very variable (more than 2000 µg g^−1^ for Ca and K or less than 1 ng g^−1^ for others). The results were similar to those reported by other European Union (EU) studies, but different from those recovered outside the EU. These differences should be eliminated to guarantee the right to health worldwide. The concentrations of Cd, Mn, Ni, Pb, and Zn in the infant formulas studied were always below the considered limits. However, it is important to check for potentially toxic elements in infant formulas to protect the health of this sensitive population. The data found in this study could be used as benchmark data for future research.

## 1. Introduction

Food safety is an issue of great interest for public health, as demonstrated by the robust regulation, the World Health Organization (WHO) recommendations and many scientific research types in the field [1,2,3]. However, due to the complexity of the topic, food safety is still the subject of toxicological and health threat evaluations, both for microbiological and chemical risks. Particular and specific attention is given to infant feeding [4]. Scientific institutions and associations that deal with children’s health emphasize the importance of breastfeeding as long as possible [5,6,7]. WHO strongly recommends exclusive breastfeeding for the first six months of life to ensure optimal growth, development, and health [8]. Breast milk provides all the nutrients, vitamins and minerals. At the same time, breastfeeding satisfies the emotional and psychological needs of the newborn and creates a special bond between mother and child with positive repercussions for life [9]. When breastfeeding is not possible, infant formulas are the essential alternative to support the newborn growth [8]. They are defined as food products intended for feeding infants in the first months of life and are prepared to satisfy, by itself, the nutritional needs of infants until the introduction of adequate complementary nutrition [10]. These formulas are the only products that can be used as substitutes. Thus, the microbiological and chemical safety of infant formulas is essential to protect the newborn’s health, and it requires careful evaluation and specific criteria to ensure the highest quality [11]. As regards to the chemical quality, limits set for contaminants or nutrient levels recommended for the adult population cannot be extended to infants since diet, energy requirements and consumption of nutrients are entirely different. Therefore, risk assessments made for infants’ diet should be specifically dedicated. The Standard for Infant Formula and Formulas for Special Medical Purposes Intended for Infants CXS 72-1981 [12] contains provisions for essential composition, quality, and safety factors and constitutes the international reference for these products. However, in some countries, regulations have been developed by leading authorities such as the European Commission [10]; as a result, estimated product specifications may differ from country to country [13]. Specific regulations impose minimum and maximum levels for some essential elements (Na, K, Cl, Ca, P, Mg, Fe, Zn, Cu, I, Se, Mn, and F) and maximum limits for some inorganic contaminants (As, Cd, Pb, Hg, and Sn) [14]. Cd, Cr, Mn, Ni, Pb, and Zn are heavy metals of great concern because they can bio-accumulate in vital organs, persist for long periods of time in the human body, and can determine several negative outcomes for human health, already in the early stages of life [15,16,17]. The intake of Cd is closely related to adverse effects on the kidney, central nervous system, and alterations in Ca metabolism [18]. The excess Mn intake can cause significant neurotoxic effects for early brain development [19,20]. Ni, a known genotoxic and carcinogen, is toxic for hematological, immunological, neurological, pulmonary, reproductive systems [21]. Pb, even at low concentrations, affects infant cognitive and neurobehavioral development [15]. Cr and Zn are essential micronutrients but, when taken in excess, can determine negative consequences for human health [22]. Besides, currently, legislation dedicated to infant formulas prescribes limits for a few number of elements and does not take into consideration some other ones, such as Al, which was found in high concentrations in some types of formulas [23]. In these cases, it is necessary to respect the precautionary principle to ensure the maximum protection of a particularly susceptible population such as infants [24].

Due to the wide range of chemicals to be determined and the need for dedicated risk assessment, a research agenda useful both for the scientific community and food companies is the development of multi-analyte methods to provide reliable and rapid results at low costs.

The aim of the present study was to determine 40 elements in all the powdered infant formulas authorized and commercialized in Italy at the time of the study by inductively coupled plasma mass spectrometer (ICP-MS). Besides, infants’ intake of some elements of relevance for human health (Cd, Cr, Mn, Ni, Pb and Zn) was estimated to assess the possible related health risks.

## 2. Materials and Methods

### 2.1. Sample Collection and Preparation

The selected powdered infant formulas were included in the National Register of products defined by the Italian Ministry of Health and established according to the Ministerial Decree of 17 May 2016 [25]. Indeed, all infant formulas must be entered in the National Register to be marketed in Italy. At the time of the study’s conduction (April 2019), the National Register included 16 powdered and three liquid formulas. A total of 11 powdered infant formulas were collected from the local pharmacies, while five products were not available. We purchased two packs of powdered milk of each marketed product for a total of 22 packs.

Each sample of the studied formula was accurately weighed (~0.5 g) using an analytical balance (Europe 60; Gibertini Elettronica, Milan, Italy) and single-use weighing boats. Each sample was placed in a polyethylene tube and carried out a “weighted by difference”; that is, the first weight of the sample subtracted from the weight of what is left in the weighing boats. This process was carried out twice, and the tubes were labeled as A and B. Each tube was analyzed in triplicate and all the results from the two packs were used to calculate the final mean of each studied formula.

In a second phase, 0.25 mL of 30% H_2_O_2_ super-pure and 0.5 mL of 67% HNO_3_ super-pure (Promochem, LGC Standards GmbH, Wesel, Germany) were added to each test tube using micropipettes (Gilson, Middleton, WI, USA), changing the disposable tip each time. The samples were digested using a water bath (WB12; Argo Lab, Modena, Italy) at 95 °C for 20 min [26]. Then, the test tubes were left to cool, and the content was diluted to 10 mL with deionized water. Samples were then filtered using a 0.45 μm microcellulose filter (GVS Life Sciences, GVS North America, Sanford, ME, USA) pre-washed with 1% (*v*/*v*) HNO_3_. About 2 mL were discarded for each sample, while the remaining volume was put in another test tube and then inserted into the test holder for analytical determinations.

The method accuracy was checked through spike samples standard reference material (SRM 1954; National Institute of Standards and Technology, Gaithersburg, MD, USA), and European reference materials (ERM^®^-BD150 and ERM^®^-BD151, Joint Research Centre, Institute for Reference Materials and Measurements, Geel, Belgium), used as previously reported [27,28,29]. Briefly, the certified material was reconstituted according to the manufacturer’s recommendations and analyzed after calibration. There was a good agreement between the obtained results and certified values, with trueness bias percentages ranging from −7 to 8% and precision <5%. Acceptable recoveries ranged from 91 to 107% and precision <15% were obtained by spiked cow milk samples. Method blanks were prepared by diluting a proper amount of 1% (*v*/*v*) HNO_3_ in polyethylene tubes.

### 2.2. Instrumentation

An ICP-MS (820-MS Bruker, Bremen, Germany), equipped with a glass nebulizer (0.4 mL min^−1^; MicroMistTM) and a collision reaction interface (CRI) with He and H_2_ (99.9995% purity; SOL Spa, Monza, Italy) was used for multi-element analysis.

A solution of Y (5 μg L^−1^ from 1000 ± 2 mg L^−1^; Panreac Quimica, Barcelona, Spain), Sc, Rh, Th and In (10 μg L^−1^ from 1000 ± 2 mg L^−1^, Panreac Quimica, Barcelona, Spain) in 2% (*v*/*v*) HNO_3_ [29] was used as internal standards.

### 2.3. Quality Assurance

The limits of detection (LODs) and quantification (LOQs) were calculated, respectively, as three and ten times the blank sample’s standard deviation (six replicates). For each element, arithmetic means (AMs) and standard deviations (SDs) were calculated (three replicates); when the result was lower than the LOD, it was considered LOD/2. When the single element results were below the LOD for more than 30% of samples, the element was excluded from subsequent elaboration.

### 2.4. Risk Evaluation

The daily intake for each studied heavy metal (Cd, Cr, Mn, Ni, Pb, and Zn) was estimated considering the concentration of the metal obtained from the analysis of the samples, the average daily/weekly intake of the formula, and the average body weight (bw) for males and females separately, and considering different time intervals from birth to 6 months old according to the nutrition requirements specific for each period of life.

The metal concentration was directly derived from the analysis as the means of the values obtained from all the analyzed formulas, as previously performed by Eticha et al., 2018 [30]. Daily doses were calculated using the infant’s feed tables. The average bw was determined according to the child growth standards charts developed by WHO [31], considering the 50th percentile of the weight for males and females at 1st week (for the period of life of 0–2 weeks), 3rd week (for 2–4 weeks), 1st month (for 2 months), 4th month (for 4 months) and 6th month (for 6 months). The daily/weekly intake for each metal was calculated by the following equation:

$$Daily\;intake\;\left(µg\;kg^{-1}\;bw\right)=\frac{\mathrm{Cm}\times \mathrm{EI}}{\mathrm{bw}}$$

Cm is the mean concentration of each studied metal in the formulas, expressed as µg g^−1^;

EI is the daily or weekly estimated intake of formulas expressed as g;

bw is the body weight expressed as kg.

Finally, each considered metal’s health risk index was calculated as a percentage of its safety limit. The safety limits were as follows: for Cd, the European Food Safety Authority (EFSA) Panel on Contaminants in the Food Chain indicates a provisional tolerable weekly intake (PTWI) of 2.5 μg kg^−1^ bw/week [32,33]; for Pb, the Joint FAO/WHO Expert Committee on Food Additives (JECFA) reports a PTWI equal to 3.5 μg kg^−1^ bw/week [34]; for Zn, the Scientific Committee on Food (SCF) indicates a tolerable upper limit of 7 mg/day [35]. A total of 300 and 2.8 μg kg^−1^ bw/day were recommended by the EFSA as the provisional tolerable daily intake (PTDI) for Cr and Ni, respectively [36,37].

As regards to Mn, we considered the current EU and French regulatory minima and maxima values for infant and follow-on formulas, that stipulated a minimum content of 1 μg of Mn/100 kcal and a maximum content of 100 μg of Mn/100 kcal [38,39]. In order to compare our results with regulatory values, we converted the minimum and maximum level from μg kcal^−1^ to μg g^−1^ by calculating the mean kcal content of 1 g of all the studied formulas, that resulted equal to 5.04 kcal per gram of formula; thus, the regulatory limits resulted 0.0504 and 5.04 μg Mn g^−1^ formula, respectively.

## 3. Results

Table 1 shows the limits of determination (LODs) and the percentages below the LOD for the 40 elements analyzed in this study.

Be, B, Al, Zr, Nb, Sb, Te, W, V, Cr, and As were excluded from the following analysis because, in the 11 infant formulas considered in the present study, these elements were detected in concentrations lower than the LOD in more than 30% of cases.

The descriptive statistics (AM and the SD, median and interquartile range—IQR) of the remaining element concentrations were calculated for each formula and are reported in Table 2, Table 3, Table 4, Table 5 and Table 6.

The values obtained by the analytical determinations resulted to be extremely variable, ranging between more than 2000 µg g^−1^ for Ca and K and less than 1 ng g^−1^ for elements such as Tl and U. Besides, we recovered different concentrations for each element in the 11 considered formulas.

**Figure 1:**
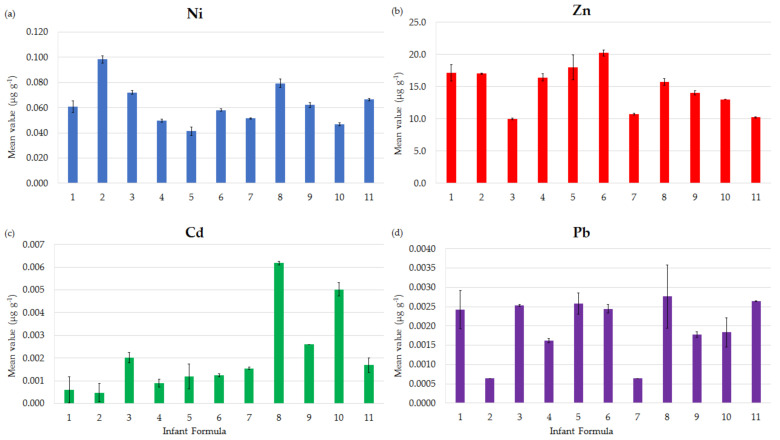
Mean values of the concentration, expressed in µg g^−1^ for each formula, of (**a**) Ni, (**b**) Zi, (**c**) Cd and (**d**) Pb. The black line on each bar represents the standard deviation.

Figure 1 shows the mean concentrations, expressed as µg g^−1^ for each formula, of the elements selected for the risk assessment (Ni, Zn, Cd, Pb). Cr and Mn are not reported in Figure 1 because more than 30% of the values of Cr were below the LOD and the risk evaluation for Mn was performed comparing its concentrations with the minima and maxima values stipulated by EU and French regulations.

The highest levels of the selected elements were detected in formula 2 for Ni (0.098 ± 0.003 µg g^−1^), in formula 6 for Zn (20.2 ± 0.5 µg g^−1^), and in formula 8 for Cd and Pb (respectively 0.0062 ± 0.0001 µg g^−1^ and 0.0028 ± 0.0008 µg g^−1^).

The concentrations of the daily/weekly intake of Cd, Ni, Pb, and Zn, calculated separately for males and females, are reported in Table 7.

Zn shows the highest daily intake (359 µg kg^−1^ bw/day for males and 381 for females at 0–2 weeks), while Cd and Pb intake at 6 months was 0.22 µg kg^−1^ bw/week for males and 0.23 for females.

Table 8 reports daily (Ni and Zn) and weekly (Cd and Pb) safety limits and the related percentage health risk indexes.

The highest health risk indexes were obtained for Ni, while the indexes for Cd always resulted lower than 15%, those relating to Pb always lower than 10% and those for Zn about 5%.

**Figure 2:**
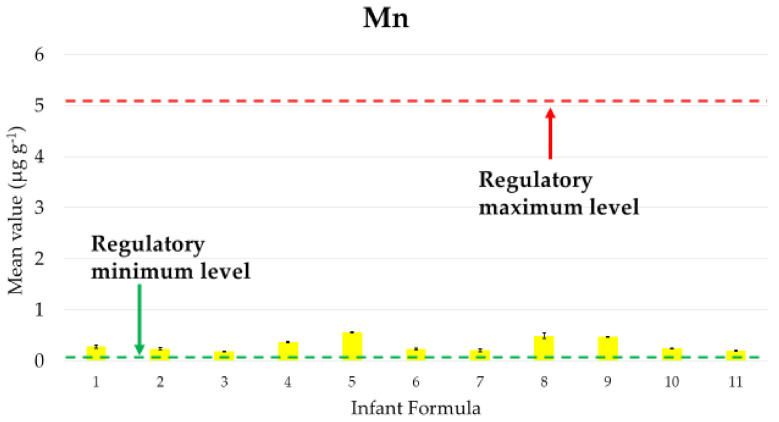
Mean values of the concentration, expressed in µg g^−1^ for each formula, of Mn. The black line on each bar represents the standard deviation; the green and red lines represent, respectively, the regulatory minimum and maximum levels.

Figure 2 shows the mean concentrations of Mn in each of the 11 studied formulas together with the minima and maxima levels prescribed by EU and French regulations.

The Mn mean concentrations found in the studied formulas were always higher than the requested minimum level and much lower than the fixed maximum level in any product.

## 4. Discussion

The present study was conducted on 11 powdered formulas commercialized in Italy. A specific risk evaluation was performed for some toxic metals (Cd, Mn, Ni, Pb, and Zn; Cr, initially considered for the risk assessment, was lower than the LOD in more than 30% of samples and, therefore, was excluded from this specific evaluation).

The first relevant result was related to the concentrations of the studied elements in the 11 formulas: 11 out of the 40 analytes (Be, B, Al, Zr, Nb, Sb, Te, W, V, Cr, and As) were lower than the LOD in more than 30% of cases. Among these elements, the levels of Al were the most surprising result because it was found to be lower than the LOD in 73% of the determinations. This finding is very positive from a food safety point of view because a chronic intake of Al via ingestion may negatively impact human health in the early stages of life [40]. Our results widely differ from those recovered previously, reporting Al levels as always higher than the LOD [41,42]. The origin of Al contamination in infant formula is not completely clear, but it probably derives from ingredients, packaging, and processing. Our result provides an indication of the improvement in the quality of food raw materials and of the production processes and food contact materials. Regarding other elements, the analytes’ concentrations reported on the infant formula labels were very similar to those recovered by the present study, confirming its accuracy and usefulness for powdered infant formulas. Besides, in our knowledge, this is the first time that 40 elements were determined in powdered infant formulas. Thus, our results are original and could be used as benchmark data for future research because infant formulas play an essential role in those specific situations when breastfeeding is not possible. Indeed, about 16% of infants are not breastfed, while up to 75% are fed with both breast milk and formulas [43].

Another important finding is related to Cd, Mn Ni, Pb, and Zn, which have been deeply studied due to their toxicity. In particular, Zn, Cd, and Pb levels ranged from 10.2 to 20.2 µg g^−1^, from 0.001 to 0.006 µg g^−1^, and from 0.0006 to 0.0026 µg g^−1^, respectively. These results were in line with those reported by another study conducted in the European Union (EU) [44], which recovered Zn, Cd, and Pb range concentrations, respectively, equal to 36.5–52.3 µg g^−1^, 0.0033–0.0045 µg g^−1^ and 0.0082–0.0439 µg g^−1^. Similarly, Bargellini et al. [45] reported concentrations for Cd, Pb, and Zn found in infant formula samples in the same order of magnitude. In contrast, other studies performed in countries outside the EU recovered very different levels. For example, a study in the field conducted in 2018 [30] on some infant formulas sold in Ethiopia reported a range concentration of Zn and Pb equal to 27.9–71.5 µg g^−1^ and <LOD–0.103 µg g^−1^, respectively, while Cd concentrations were always <LOD. These differences in elemental concentrations all over the countries were also evidenced by other researches in China and Pakistan [46,47]. Likewise, similarities between the elemental concentrations found in formulas marketed in EU countries and differences with the levels reported for the formulas sold in countries outside EU are due to differences in raw materials, processing and packaging, and regulations. It should be essential to harmonize procedures and regulations worldwide to guarantee the same right to health, one of the internationally agreed human rights recognized by WHO.

As regards to Mn, we found mean concentrations always lower than the maximum level prescribed by EU and French regulations for infant formulas. This result is in line with those reported for the levels of Mn recovered in 17 powdered infant or follow-on formulas purchased in France and 14 infant formula products purchased in the United States. In contrast, a soy-based infant formula and an amino acid-based medical infant formula exceeded 100 μg of Mn/100 kcal [19,20].

In the present study, we also estimated the intake of Cd, Ni, Pb, and Zn and assessed the related risk, according to gender and different periods from birth to 6 months of life; for this purpose, the estimated daily/weekly intake of Cd, Ni, Pb and Zn and their percentage health risk indexes were calculated. The results showed that the heavy metals intake from infant formulas was not so relevant and always lower than the safety limit. However, some heavy metals’ ingestion occurs via powdered infant formula and this is not negligible, especially considering that the study population is highly susceptible. Besides, contaminants such as Zn, Cd, Ni, and Pb are ubiquitous in the environment and can also be assumed through inhalation and skin absorption. Thus, the amount taken through infant formula represents just a part of the total intake, and it should be added to the quantities introduced into the body with air and with dermal contact. In addition, the contribution of the minerals and potentially toxic elements present in the water used for the reconstitution of the powdered formulas should be considered.

Similar risk evaluations have also been performed for breast milk, evidencing detectable levels of potential toxic elements such as Pb, Cd, Cu, As, Zn [27,48,49]. All authors, however, agree on the great variability of the concentration of these elements due to mothers’ dietary habits, lifestyles, occupational exposure, urban pollution exposure, lactation stage, etc. These interfering factors do not influence the elemental levels in infant formula products.

This study presents some limitations. First of all, the number of infant formulas was limited to 11, but these were the formulas authorized and commercialized in Italy when we collected the samples. Besides, we determine a very high number of elements for each studied formula with a unique analysis. Secondly, we estimated the dose of some toxic elements assumed by newborns through infant formula using the mean concentrations based on the analysis of only one lot for each formula. However, the manufacturing process of such products is strictly regulated by the specific international regulations (The Standard for Infant Formula and Formulas for Special Medical Purposes Intended for Infants CXS 72-1981); thus, differences among lots are necessarily below the acceptable quality limit. Finally, we assumed a theoretical weight; however, we performed the evaluation by considering different periods between the birth to the sixth month of life, and it was performed separately for males and females.

## 5. Conclusions

A determination of 40 elements in powdered infant formulas was performed. The concentration of the elements in 11 infant formulas authorized and commercialized in Italy demonstrated levels for some elements similar to those reported by other EU studies but different from those performed outside the EU. It is essential to eliminate these differences to guarantee the right to health for all the newborns all over the word. The specific risk assessment performed for Cd, Mn, Ni, Pb, and Zn demonstrated that the concentrations of these elements in the studied infant formulas were always below the considered limits; however, this issue must be constantly considered because the amount of potentially toxic elements assumed with infant formulas is just a part of all the quantity assumed (adding to the amount assumed via inhalation and dermal contact).

## Figures and Tables

**Table 1:** Selection of isotopes, limit of determination (LOD) expressed as µg g^−1^ formula and percentage of values lower than LOD for each element.

Element	LOD	% <LOD
^7^Li	0.00005	0
^9^Be	0.0001	82
^11^B	0.6	100
^23^Na	4	0
^24^Mg	0.3	0
^27^Al	0.01	73
^28^Si	1	0
^31^P	4	0
^39^K	5	0
^44^Ca	3	0
^49^Ti	0.002	0
^59^Co	0.0003	0
^60^Ni	0.02	0
^65^Cu	0.01	0
^66^Zn	0.1	0
^71^Ga	0.0004	0
^85^Rb	0.001	0
^88^Sr	0.01	0
^90^Zr	0.002	59
^93^Nb	0.01	100
^98^Mo	0.004	0
^112^Cd	0.0004	9
^118^Sn	0.0002	0
^121^Sb	0.01	77
^125^Te	0.003	91
^133^Cs	0.00005	0
^137^Ba	0.01	0
^139^La	0.00003	0
^140^Ce	0.0001	0
^182^W	0.002	50
^205^Tl	0.0001	5
^208^Pb	0.001	18
^209^Bi	0.001	23
^238^U	0.00003	0
^51^V	0.002	36
^52^Cr	0.01	32
^55^Mn	0.004	0
^56^Fe	0.1	0
^75^As	0.01	77
^76^Se	0.01	0

**Table 2:** Descriptive statistics of Li, Na, Mg, Si, P and K calculated on a total of 12 determinations for each formula and expressed as µg g^−1^.

Infant Formula	Li		Na		Mg		Si		P		K	
	AM ± SD	Median IQR	AM ± SD	Median IQR	AM ± SD	Median IQR	AM ± SD	Median IQR	AM ± SD	Median IQR	AM ± SD	Median IQR
1	0.0060 ± 0.001	0.00603 0.00581–0.00626	635 ± 37	634.8 621.6–648.0	204 ± 14	204.2 199.4–209.0	22.0 ± 1.3	22.03 21.57–22.50	770 ± 50	770.2 752.6–787.9	2439 ± 154	2438.6 2384.1–2493.1
2	0.0057 ± 0.0001	0.00574 0.00573–0.00575	519 ± 14	518.9 514.2–523.7	311 ± 1	311.2 311.1–311.3	19.4 ± 1.0	19.40 19.04–19.75	598 ± 14	597.5 592.7–602.3	2058 ± 58	2058.1 2037.8–2078.5
3	0.0037 ± 0.0001	0.00369 0.00364–0.00374	687 ± 25	687.1 678.3–695.9	133 ± 3	133.4 132.3–134.5	19.7 ± 0.9	19.66 19.36–19.97	906± 23	906.2 898.0–914.5	2085 ± 53	2085.2 2066.5–2103.9
4	0.0076 ± 0.0003	0.00758 0.00747–0.00769	583 ± 19	582.7 576.2–589.2	161 ± 4	160.9 159.5–162.3	22.5 ± 0.3	22.54 22.42–22.66	699 ± 13	699.2 694.7–703.7	2518 ± 54	2517.5 2498.5–2536.5
5	0.0098 ± 0.0008	0.00985 0.00955–0.01014	715 ± 73	715.3 689.4–741.1	281 ± 37	281.0 268.0–294.1	18.9 ± 1.2	18.91 18.50–19.33	544 ± 46	543.5 527.3–559.6	2578 ± 210	2578.1 2505.3–2651.0
6	0.0074 ± 0.0002	0.00741 0.00736–0.00747	619 ± 20	618.6 611.6–625.5	167 ± 5	167.4 165.7–169.1	18.4 ± 0.3	18.43 18.33–18.52	817 ± 8	817.2 814.2–820.2	2169 ± 35	2169.1 2156.6–2181.5
7	0.0055 ± 0.0001	0.00547 0.00546–0.00549	535 ± 6	535.0 532.8–537.2	137 ± 1	136.7 136.6–136.8	18.2 ± 0.5	18.15 17.97–18.33	692 ± 12	691.9 687.8–696.0	1929 ± 2	1928.7 1928.0–1929.4
8	0.0145 ± 0.0001	0.01451 0.01452–0.01455	511 ± 1	511.3 511.0–511.6	156 ± 1	156.1 155.8–156.3	20.0 ± 0.1	20.02 19.98–20.06	582 ± 13	581.8 577.1–586.6	2395 ± 12	2394.8 2390.4–2399.2
9	0.0116 ± 0.0004	0.01157 0.01144–0.01171	533 ± 10	532.4 529.0–535.9	197 ± 4	196.5 195.0–198.1	19.2 ± 0.6	19.20 18.98–19.42	865 ± 14	865.1 860.1–870.2	2607 ± 56	2607.0 2587.3–2626.8
10	0.0045 ± 0.0001	0.00451 0.00448–0.00454	494 ± 5	493.7 491.9–495.5	154 ± 2	153.5 152.8–154.1	18.0 ± 0.3	18.03 17.94–18.12	632 ± 11	631.6 627.7–635.4	2410 ± 42	2410.6 2395.9–2425.3
11	0.0033 ± 0.0001	0.00333 0.00332–0.00335	458 ± 5	457.6 455.9–459.2	129 ± 2	128.5 127.7–129.3	18.4 ± 0.1	18.36 18.34–18.38	759 ± 13	758.9 754.2–763.6	2028 ± 26	2028.0 2018.9–2037.2

AM = arithmetic mean; SD = standard deviation; IQR = interquartile range.

**Table 3:** Descriptive statistics of Ca, Ti, Co, Cu, Ga and Rb calculated on a total of 12 determinations for each formula and expressed as µg g^−1^.

Infant Formula	Ca		Ti		Co		Cu		Ga		Rb	
	AM ± SD	Median IQR	AM ± SD	Median IQR	AM ± SD	Median IQR	AM ± SD	Median IQR	AM ± SD	Median IQR	AM ± SD	Median IQR
1	2729 ± 36	2729.0 2716.3–2741.8	0.105 ± 0.005	0.1054 0.1035–0.1073	0.012 ± 0.001	0.0118 0.0115–0.0120	1.33 ± 0.08	1.329 1.299–1.358	0.004 ± 0.001	0.0036 0.0033–0.0038	2.92 ± 0.05	2.922 2.906–2.939
2	2144 ± 14	2143.9 2138.8–2148.9	0.098 ± 0.001	0.0977 0.0974–0.0979	0.010 ± 0.001	0.0097 0.0096–0.0097	1.731 ± 0.001	1.731 1.730–1.732	0.003 ± 0.001	0.0027 0.0025–0.0029	2.48 ± 0.10	2.482 2.448–2.517
3	2687 ± 16	2686.5 2680.8–2692.1	0.092 ± 0.006	0.0922 0.0903–0.0942	0.014 ± 0.001	0.0144 0.0142–0.0146	1.04 ± 0.03	1.036 1.027–1.046	0.003 ± 0.001	0.0032 0.0029–0.0035	2.93 ± 0.01	2.930 2.928–2.933
4	2119 ± 31	2118.6 2107.5–2129.6	0.089 ± 0.001	0.0893 0.0890–0.0896	0.011 ± 0.001	0.0113 0.0112–0.0115	1.40 ± 0.05	1.395 1.377–1.413	0.005 ± 0.002	0.0047 0.0040–0.0054	2.51 ± 0.57	2.510 2.309–2.711
5	1783 ± 20	1783.3 1776.2–1790.4	0.112 ± 0.013	0.1125 0.1080–0.1170	0.008 ± 0.001	0.0081 0.0079–0.0083	1.64 ± 0.20	1.643 1.574–1.713	0.003 ± 0.001	0.0027 0.0026–0.0028	0.60 ± 0.02	0.595 0.590–0.601
6	2517 ± 41	2516.9 2502.3–2531.5	0.099 ± 0.002	0.0991 0.0983–0.0998	0.010 ± 0.001	0.0104 0.0104–0.0105	1.05 ± 0.03	1.047 1.038–1.056	0.003 ± 0.001	0.0031 0.0030–0.0032	2.22 ± 0.13	2.217 2.172–2.263
7	2423 ± 56	2423.2 2403.6–2442.9	0.092 ± 0.004	0.0920 0.0907–0.0933	0.010 ± 0.001	0.0096 0.0095–0.0097	0.800 ± 0.008	0.800 0.797–0.803	0.003 ± 0.001	0.0033 0.0032–0.0034	2.26 ± 0.16	2.259 2.204–2.314
8	2478 ± 2	2477.5 2476.9–2478.2	0.122 ± 0.004	0.1217 0.1202–0.1231	0.010 ± 0.001	0.0100 0.0099–0.0101	1.11 ± 0.01	1.111 1.107–1.116	0.002 ± 0.001	0.0016 0.0015–0.0018	0.39 ± 0.01	0.386 0.383–0.389
9	2814 ± 17	2813.6 2807.6–2819.5	0.103 ± 0.004	0.1028 0.1014–0.1043	0.013 ± 0.001	0.0133 0.0132–0.0134	1.35 ± 0.03	1.351 1.339–1.364	0.007 ± 0.001	0.0068 0.0066–0.0071	7.27 ± 0.18	7.268 7.204–7.332
10	2303 ± 28	2302.5 2292.5–2312.5	0.081 ± 0.002	0.0815 0.0807–0.0823	0.009 ± 0.001	0.0093 0.0092–0.0093	1.13 ± 0.03	1.131 1.122–1.141	0.004 ± 0.001	0.0043 0.0041–0.0044	4.02 ± 0.10	4.016 3.980–4.052
11	2505 ± 22	2505.3 2497.6–2512.9	0.084 ± 0.002	0.0836 0.0830–0.0843	0.014 ± 0.001	0.0143 0.0140–0.0146	1.13 ± 0.02	1.126 1.119–1.133	0.004 ± 0.001	0.0038 0.0035–0.0040	3.97 ± 0.20	3.968 3.896–4.040

AM = arithmetic mean; SD = standard deviation; IQR = interquartile range.

**Table 4:** Descriptive statistics of Sr, Mo, Sn, Cs, Ba and La calculated on a total of 12 determinations for each formula and expressed as µg g^−1^.

Infant Formula	Sr		Mo		Sn		Cs		Ba		La	
	AM ± SD	Median IQR	AM ± SD	Median IQR	AM ± SD	Median IQR	AM ± SD	Median IQR	AM ± SD	Median IQR	AM ± SD	Median IQR
1	1.59 ± 0.02	1.590 1.583–1.598	0.172 ± 0.002	0.1717 0.1709–0.1724	0.0969 ± 0.0874	0.09694 0.06603–0.12785	0.0190 ± 0.0009	0.01898 0.01865–0.01931	0.214 ± 0.001	0.2138 0.2136–0.2140	0.0063 ± 0.0044	0.00632 0.00476–0.00789
2	1.28 ± 0.04	1.284 1.268–1.300	0.076 ± 0.0003	0.0759 0.0758–0.0760	0.0484 ± 0.0177	0.04837 0.04212–0.05461	0.0070 ± 0.0001	0.00705 0.00701–0.00708	0.141 ± 0.003	0.1407 0.1395–0.1419	0.0029 ± 0.0001	0.00289 0.00285–0.00293
3.	1.02 ± 0.01	1.019 1.017–1.021	0.189 ± 0.006	0.1886 0.1867–0.1906	0.0028 ± 0.0012	0.00282 0.00239–0.00326	0.0038 ± 0.0001	0.00382 0.00381–0.00383	0.121 ± 0.008	0.1205 0.1178–0.1232	0.0011 ± 0.0001	0.00107 0.00105–0.00108
4	1.12 ± 0.24	1.115 1.029–1.201	0.107 ± 0.017	0.1075 0.1014–0.1135	0.0106 ± 0.0038	0.01058 0.00922–0.01194	0.0052 ± 0.0002	0.00519 0.00512–0.00525	0.207 ± 0.021	0.2073 0.1998–0.2149	0.0036 ± 0.0001	0.00356 0.00354–0.00358
5	0.529 ± 0.018	0.529 0.523–0.536	0.115 ± 0.001	0.1149 0.1145–0.1154	0.0237 ± 0.0051	0.02371 0.02190–0.02551	0.0015 ± 0.0001	0.00151 0.00147–0.00154	0.111 ± 0.011	0.1109 0.1069–0.1148	0.0006 ± 0.0002	0.00061 0.00055–0.00068
6	1.44 ± 0.11	1.438 1.401–1.475	0.242 ± 0.018	0.2422 0.2360–0.2484	0.0032 ± 0.0007	0.00319 0.00294–0.00345	0.0074 ± 0.0002	0.00745 0.00736–0.00754	0.140 ± 0.002	0.1397 0.1391–0.1402	0.0002 ± 0.0001	0.00025 0.00023–0.00026
7	1.44 ± 0.12	1.440 1.397–1.482	0.276 ± 0.037	0.2763 0.2633–0.2893	0.0054 ± 0.0049	0.00539 0.00367–0.00712	0.0079 ± 0.0003	0.00794 0.00783–0.00805	0.179 ± 0.018	0.1788 0.1725–0.1851	0.0010 ± 0.0002	0.00097 0.00091–0.00103
8	0.827 ± 0.015	0.827 0.822–0.833	0.056 ± 0.004	0.0564 0.0551–0.0578	0.0009 ± 0.0001	0.00094 0.00093–0.00096	0.0057 ± 0.0003	0.00568 0.00558–0.00578	0.074 ± 0.002	0.0739 0.0730–0.0747	0.0211 ± 0.0002	0.02110 0.02104–0.02117
9	2.34 ± 0.04	2.340 2.326–2.354	0.124 ± 0.005	0.1235 0.1217–0.1254	0.0003 ± 0.0001	0.00029 0.00028–0.00030	0.0145 ± 0.0003	0.01455 0.01443–0.01466	0.420 ± 0.003	0.4198 0.4188–0.4208	0.0018 ± 0.0001	0.00185 0.00181–0.00189
10	1.08 ± 0.01	1.077 1.073–1.081	0.195 ± 0.002	0.1948 0.1940–0.1955	0.0007 ± 0.0001	0.00071 0.00070–0.00072	0.0127 ± 0.0002	0.01274 0.01269–0.01280	0.180 ± 0.001	0.1805 0.1800–0.1810	0.0040 ± 0.0001	0.00401 0.00400–0.00402
11	0.78 ± 0.04	0.781 0.767–0.796	0.146 ± 0.003	0.1458 0.1446–0.1470	0.0269 ± 0.0003	0.02688 0.02677–0.02700	0.0043 ± 0.0002	0.00434 0.00428–0.00440	0.137 ± 0.002	0.1369 0.1361–0.1377	0.0022 ± 0.0001	0.00216 0.00213–0.00219

AM = arithmetic mean; SD = standard deviation; IQR = interquartile range.

**Table 5:** Descriptive statistics of Ce, Tl, and Bi calculated on a total of 12 determinations for each formula and expressed as µg g^−1^.

Infant Formula	Ce		Tl		Bi	
	AM ± SD	Median IQR	AM ± SD	Median IQR	AM ± SD	Median IQR
1	0.0056 ± 0.0054	0.00560 0.00368–0.00753	0.00042 ± 0.00006	0.000418 0.000394–0.000441	0.0040 ± 0.0005	0.00404 0.00388–0.00420
2	0.0011 ± 0.0001	0.00114 0.00111–0.00116	0.00069 ± 0.00001	0.000685 0.000682–0.000688	0.0035 ± 0.0002	0.00347 0.00340–0.00353
3.	0.0006 ± 0.0001	0.00063 0.00061–0.00065	0.00061 ± 0.00004	0.000606 0.000589–0.000623	0.0014 ± 0.0001	0.00141 0.00127–0.00144
4	0.0013 ± 0.0001	0.00129 0.00126–0.00133	0.00020 ± 0.00012	0.000199 0.000155–0.000242	0.0013 ± 0.0001	0.00138 0.00126–0.00127
5	0.0009 ± 0.0001	0.00093 0.00091–0.00095	0.00084 ± 0.00007	0.000838 0.000812–0.000863	0.0004 ± 0.0001	0.00042 0.00042–0.00042
6	0.0003 ± 0.0001	0.00026 0.00026–0.00027	0.00055 ± 0.00013	0.000551 0.000505–0.000598	0.0023 ± 0.0003	0.00231 0.00218–0.00243
7	0.0009 ± 0.0001	0.00086 0.00085–0.00087	0.00054 ± 0.00004	0.000544 0.000529–0.000559	0.0022 ± 0.0002	0.00219 0.00212–0.00225
8	0.0067 ± 0.0003	0.00670 0.00659–0.00681	0.00109 ± 0.00004	0.001086 0.001071–0.001101	0.0123 ± 0.0001	0.01227 0.01224–0.01230
9	0.0010 ± 0.0002	0.00101 0.00094–0.00107	0.00038 ± 0.00001	0.000383 0.000377–0.000389	0.0004 ± 0.0001	0.00042 0.00042–0.00042
10	0.0017 ± 0.0001	0.00172 0.00167–0.00176	0.00131 ± 0.00007	0.001308 0.001280–0.001336	0.0017 ± 0.0001	0.00171 0.00171–0.00172
11	0.0010 ± 0.0001	0.00101 0.00099–0.00104	0.00055 ± 0.00001	0.000550 0.000549–0.000551	0.0008 ± 0.0005	0.00076– 0.00059–0.00093

AM = arithmetic mean; SD = standard deviation; IQR = interquartile range.

**Table 6:** Descriptive statistics of U, Fe and Se calculated on a total of 12 determinations for each formula and expressed as µg g^−1^.

Infant Formula	U		Fe		Se	
	AM ± SD	Median IQR	AM ± SD	Median IQR	AM ± SD	Median IQR
1	0.00074 ± 0.00001	0.000740 0.000739–0.000741	31.4 ± 0.3	31.40 31.28–31.52	0.151 ± 0.007	0.1513 0.14893–0.15359
2	0.00068 ± 0.00001	0.000681 0.000675–0.000687	32.2 ± 0.7	32.21 31.96–32.46	0.143 ± 0.014	0.1431 0.13824–0.14787
3	0.00127 ± 0.00012	0.001272 0.001231–0.001312	28.6 ± 0.2	28.62 28.56–28.67	0.147 ± 0.018	0.1468 0.14048–0.15304
4	0.00032 ± 0.00001	0.000318 0.000305–0.000331	32.9 ± 0.2	32.88 32.80–32.96	0.088 ± 0.005	0.0885 0.08680–0.09018
5	0.00385 ± 0.00001	0.003854 0.003850–0.003858	28.1 ± 0.6	28.11 27.90–28.31	0.198 ± 0.0010	0.1980 0.19763–0.19834
6	0.00053 ± 0.00001	0.000530 0.000516–0.000545	27.0 ± 0.3	27.00 26.90–27.11	0.139 ± 0.024	0.1390 0.13034–0.14758
7	0.00159 ± 0.00111	0.001585 0.001193–0.001978	23.9 ± 1.4	23.98 23.49–24.48	0.111 ± 0.029	0.1106 0.10043–0.12069
8	0.00566 ± 0.00020	0.005657 0.005585–0.005730	24.3 ± 0.1	24.23 24.18–24.28	0.158 ± 0.026	0.1576 0.14834–0.16679
9	0.00204 ± 0.00037	0.002043 0.001913–0.002172	23.6 ± 1.1	23.57 23.20–23.95	0.155 ± 0.003	0.1549 0.15393–0.15591
10	0.00490 ± 0.00029	0.004904 0.004802–0.005007	21.6 ± 0.6	21.63 21.44–21.83	0.130 ± 0.004	0.1304 0.12902–0.13178
11	0.00084 ± 0.00001	0.000843 0.000842–0.000844	34.0 ± 0.3	33.96 33.85–34.06	0.159 ± 0.005	0.1591 0.15737–0.16079

AM = arithmetic mean; SD = standard deviation; IQR = interquartile range.

**Table 7:** Daily intake of Ni and Zn and weekly intake of Cd and Pb for infants from 0 to 6 months, separately for males (M) and females (F).

Age	Amount of Infant Formula (g/day g/week)	Weight (kg)		Ni µg kg^−1^ bw/day		Zn µg kg^−1^ bw/day		Cd µg kg^−1^ bw/week		Pb µg kg^−1^ bw/week	
		M	F	M	F	M	F	M	F	M	F
0–2 weeks	85 595	3.5	3.3	1.50	1.60	359	381	0.34	0.36	0.34	0.36
2–4 weeks	95 665	4.1	3.8	1.44	1.55	343	370	0.32	0.35	0.32	0.35
2 months	105 735	5.4	5.0	1.20	1.30	288	311	0.27	0.29	0.27	0.29
4 months	130 910	7.0	6.4	1.15	1.26	275	301	0.26	0.28	0.26	0.28
6 months	122 854	7.9	7.3	0.96	1.04	229	247	0.22	0.23	0.22	0.23

**Table 8:** Percentage health risk index estimated for infants from 0 to 6 months for Ni, Zn, Cd and Pb, reported separately for males (M) and females (F).

Safety Limit	Ni 2.8 µg kg^−1^ bw/day		Zn 7 mg kg^−1^ bw/day		Cd 2.5 µg kg^−1^ bw/week		Pb 3.5 µg kg^−1^ bw/week	
Age	% Health Risk Index							
	M	F	M	F	M	F	M	F
0–2 weeks	53.6	57.1	5.13	5.44	13.6	14.4	9.71	10.2
2–4 weeks	51.4	55.4	4.89	5.28	12.8	14.0	9.14	10.0
2 months	42.9	46.4	4.11	4.44	10.8	11.6	7.71	8.28
4 months	41.1	45.0	3.96	4.29	10.4	11.2	7.40	8.00
6 months	34.3	37.1	3.26	3.53	8.8	9.2	6.28	6.57

## Data Availability

The data presented in this study are available on request from the corresponding author.
